# A Deep Learning Framework for Using Search Engine Data to Predict Influenza-Like Illness and Distinguish Epidemic and Nonepidemic Seasons: Multifeature Time Series Analysis

**DOI:** 10.2196/71786

**Published:** 2025-08-11

**Authors:** Ji Li, Xiangyu Yan, Xingjie Chu, Ying Zhang, Guoliang Liu, Lin Li, Yue Li, Xiaochun Dong, Zihan Mei, Zhengkun Liu, Jinyue Yuan, Xiaohan Sun, Chunxia Cao

**Affiliations:** 1 School of Disaster and Emergency Medicine, Tianjin University, No. 92 Weijin Road, Nankai District, Tianjin, 300072, China, 86 13512065620; 2 College of Management and Economics, Tianjin University, Tianjin, China; 3 Langfang Center for Disease Prevention and Control, Hebei, China; 4 Tianjin Centers for Disease Control and Prevention, Tianjin, China

**Keywords:** influenza, CLSTM framework, Baidu index, influenza epidemic, nonepidemic seasons, convolutional long short-term memory

## Abstract

**Background:**

The seasonal influenza epidemic poses a persistent and severe threat to global public health. Web-based search data are recognized as a valuable source for forecasting influenza or other respiratory tract infection epidemics. Current influenza prediction studies typically focus on seasonal trends in traditional monitoring data, neglecting the sensitivity of different web-based search terms to seasonal changes, thereby increasing prediction challenges.

**Objective:**

The aim of this study was to propose a deep learning framework for different influenza epidemic states based on Baidu index and percentage of influenza-like illness (ILI%).

**Methods:**

Official weekly ILI% data from 2013 to 2024 were extracted from the Chinese National Notifiable Infectious Disease Reporting System (NIDRIS). Based on the Baidu index, influenza-related search indexes were acquired for the corresponding time periods. To explore the association between influenza-related search queries and ILI%, the study conducted a cross-correlation analysis. The study period was divided into influenza epidemic and nonepidemic period. The study finally used the convolutional long short-term memory (CLSTM) network framework to predict influenza epidemics with 1‐3 weeks ahead for the all-time period and epidemic + nonepidemic period. The evaluation metrics included model stability metric, accuracy metrics, and explanatory power metric.

**Results:**

The ILI% presented a regular seasonal high incidence in China. Meanwhile, the prediction of ILI% after dividing the epidemic and nonepidemic seasons (mean absolute percentage error [MAPE]=10.730%, mean square error [MSE]=0.884, mean absolute error [MAE]=0.649, root-mean-square error [RMSE]=0.940, and *R*^2^=0.877) was better than that of the all-time period (MAPE=12.784%, MSE=1.513, MAE=0.744, RMSE=1.230, and *R*^2^=0.786). In addition, we found that the ILI% + Baidu search index predicts better than only the ILI% regardless of the time period and lag time of the study. Comparative analysis with long short-term memory (LSTM) and transformer models demonstrated that CLSTM achieved superior performance in 1 week-ahead ILI% predictions using ILI% + Baidu index data in epidemic + nonepidemic period (MAPE=11.824%, MSE=1.243, MAE=0.723, RMSE=1.115, and *R*^2^=0.827). Furthermore, CLSTM comprehensively surpasses LSTM in computational efficiency, complexity, extrapolation capability, and stability while partially outperforming transformer models.

**Conclusions:**

This study shows strong potential for influenza prediction by combining Baidu index data with traditional surveillance and specific keywords for epidemic and nonepidemic seasons. It provides a new perspective for public health preparedness. This research is expected to support early warning systems for influenza and other diseases. Future work will further optimize these models for more timely and accurate predictions, enhancing public health responses.

## Introduction

Influenza poses a persistent and severe threat to global public health [1]. The World Health Organization estimates that annual epidemics of influenza result in 1 billion infections, 3-5 million severe cases of influenza, and 300,000-650,000 deaths globally [2,3]. The National Health Commission of China reported that in 2020 and 2021, there were 1,145,278 and 668,246 influenza cases, with incidence rates of 81.5816 and 47.4008 per 100,000, respectively [4]. China has a significant global share of patients with influenza, especially during the high influenza season. Influenza in China may significantly influence global influenza trends [5]. China has a profound impact on global influenza due to its large population and its important role in global influenza surveillance, prediction, and early warning.

Currently, the main global influenza surveillance methods include monitoring influenza-like illness (ILI) and influenza virus positivity. These methods aim to capture fluctuations in patient visits and the intensity of influenza virus transmission. This provides insight into the onset, peak, and end of influenza [6,7]. Traditional surveillance methods, such as weekly data reports, play an important role in influenza surveillance but often struggle with timely early warnings due to inherent delays. In contrast, the popularity of the web and advancements in data analysis have made search engine data highly useful for identifying infectious disease outbreaks in advance.

Google Flu Trends created a new era that used Google search data to predict the percentage of influenza-like illness (ILI%) in the United States [8,9]. Thereafter, multisource electronic data including web-based search data [10-12], influenza surveillance data [10], influenza-related posts on Twitter [13-15], Wikipedia access logs [16], and electronic health records [8,17] were integrated with mathematical models to track illness activities with very good predictive results. However, the model was not stable because the influenza trend exceeded the peak of the epidemic by more than 140% in 2013 in the United States and sparked a hot discussion about the limitation of search data in infectious disease research [18]. Some studies had constructed models to improve forecasting accuracy in cities such as New York [19], Melbourne [20], and Hong Kong [21]. The field of digital epidemiology is still in an early stage, but it has begun to be used to forecast infectious disease epidemic trends, especially during the COVID-19 pandemic [22]. However, web-based search terms are vast and complex. Even within the same topic, time series correlations between search terms are weak. Current influenza prediction studies typically focus on seasonal trends in traditional monitoring data, neglecting the sensitivity of different web-based search terms to seasonal changes, thereby increasing prediction challenges.

Accordingly, this study proposed a deep learning framework for multifeature time series for different influenza epidemic states. The framework integrated Baidu index and traditional surveillance data to address the complex challenges of predicting influenza trends. In particular, the study divided the influenza epidemic states into epidemic and nonepidemic seasons. This adjustment not only allows the framework to better capture changes in influenza trends but also optimizes prediction accuracy. By integrating data from multiple sources, the framework can adjust its forecasting strategy across different influenza epidemic states.

## Methods

### Data Collection

The ILI% is the proportion of patients with ILI divided by the total number of physician visits. The ILI% based on weekly reports in Langfang, Hebei Province, China, was extracted from the Chinese National Notifiable Infectious Disease Reporting System (NIDRIS) for influenza from October 2013 to March 2024. Patients with ILI were defined as outpatients of any age with acute respiratory infection syndrome with fever ≥38 °C and cough or sore throat.

Baidu index is a statistical indicator that represents the search volume of demanding keywords or phrases based on Baidu’s search query logs, which is the largest search engine in China [23]. This study summarized influenza-related keywords that might correlate with the trend of ILI% and selected the keywords and the time range from October 2013 to March 2024. The search data were based on personal computer and personal mobile phone data.

### Descriptive Analysis

A descriptive analysis was used to show the characteristics of the current ILI% and the current search indices of different keywords on the Baidu index. This study divided the study period based on the characteristics of influenza epidemics. According to the latest influenza surveillance plan [24-26], the epidemic season is from week 40 of each year to week 13 of the following year, while the nonepidemic season is from week 14 to week 39. This division helps align with the seasonal patterns of influenza outbreaks [27]. The cross-correlation coefficient was calculated to explore the association between the influenza-related search terms data and the ILI% of the epidemic season and the nonepidemic season, respectively. The study also analyzed the correlation between the previous week’s search index (from week 1 to 4) and the ILI% of the epidemic season and the nonepidemic season. A correlation coefficient closer to 1 or –1 indicates a stronger correlation, and a correlation coefficient closer to 0 indicates a weaker correlation. The cross-correlation coefficient was calculated between each variable to observe the correlations among the variables. After performing the correlation analysis, the variables with correlation coefficients above 0.5 were selected. These variables were used in different lags to develop predicting models. This approach aimed to improve prediction accuracy based on previous studies [11,28].

### Deep Learning Framework for Multifeature Time Series

**Figure 1.**
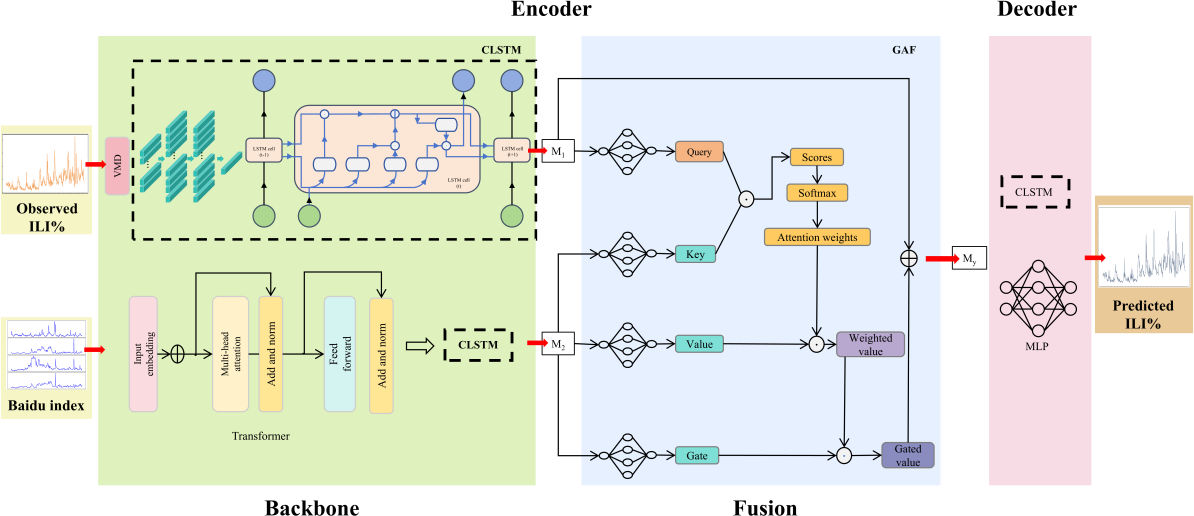
CLSTM network framework. CLSTM: convolutional long short-term memory; GAF: gated attention fusion; ILI%: percentage of influenza-like illness; LSTM: long short-term memory; MLP: multiple-layer perceptron; VMD: variational mode decomposition.

To effectively leverage the useful information in surveillance data and Baidu index data, this study proposed a deep learning framework for multifeature time series to address the challenge of predicting real-world influenza trends (Figure 1). By fully mining the inherent characteristics of data and establishing the mapping relationship between features and results, the framework could complete the task of time series prediction in a scientific and robust manner. The mathematical formulation of the framework is shown in Multimedia Appendix 1.

The models used in this study were based on an encoder-decoder framework. The input of the encoder was the data, including historical ILI% and Baidu keyword searches. The advanced ILI% forecast results were obtained from the output of the decoder. For observed ILI%, the variational mode decomposition algorithm [29] was applied to decompose original signal into multiple intrinsic mode functions. This helped remove redundant information from the observed ILI%, thereby improving prediction accuracy [30]. A time window was set and slid during each decomposition to generate new subsignals while preventing information leakage. For the Baidu index, transformer was used for the feature extraction in this study. The method used a self-attentive mechanism to capture temporal dependencies and semantic relationships in the sequences. This allowed it to extract key features that are useful for predicting ILI%. In this way, feature extraction could more accurately reflect the link between search data and influenza activity.

This study used the classical encoder-decoder framework for algorithm construction. The encoder part was divided into backbone and fusion parts. Data from different modalities were input into the encoder through 2 separate channels. The backbone part, which is a convolutional long short-term memory (CLSTM) network, was used to learn the corresponding features of each modality {M_1_, M_2_}. Gated attention fusion model was used in the fusion part. Gated attention fusion integrated features {M_1_, M_2_} from the backbone. It generated a query, key, and value using the attention mechanism and computes attention weights. Then, these weights were processed by softmax, used to weight the value. Subsequently, the weighted values were used to enable the model to selectively focus on key features through the gating mechanism, resulting in the joint feature, M_y_. The joint feature M_y_ was used as input data and sent into the decoder. In the decoder, the CLSTM module was used to decode the state vector, while the multiple-layer perceptron transformed the features generated by the CLSTM into the final ILI% prediction. In the model validation, the study introduced long short-term memory (LSTM) and transformer as baseline comparators, ensuring fair evaluation through identical training sets and assessment metrics. To further verify generalizability, the model was validated using independent datasets from Tianjin, China.

### Statistical Analysis

The ILI% surveillance data were divided into 2 parts, the training set and the test set. The training set was from the 14th week of 2013 to the 10th week of 2022, and the test set was from the 11th week of 2022 to the 13th week of 2024 for the whole study period. The training and test sets for the epidemic and nonepidemic seasons were divided according to their respective study times in a ratio of 8:2 and assigned in time order. This study used 3 types of indicators to evaluate the performance of the prediction model, including model stability metric, accuracy metrics, and explanatory power metric (Multimedia Appendix 2). Stability metric mainly uses the mean absolute percentage error (MAPE). Accuracy metrics include mean square error (MSE), root-mean-square error (RMSE), and mean absolute error (MAE). Explanatory power metric mainly uses the coefficient of determination (*R*^2^). When *R*^2^ exceeds 0.7, while MSE, RMSE, and MAE values approach 0 and MAPE falls below 20%, it indicates strong predictive performance of the model [31]. In addition to model performance, the models’ computational efficiency and computational complexity analysis were used to the models’ comparison. To evaluate computational efficiency, floating-point operations per second (FLOPs) can be used because it directly reflects the amount of computational resources and processing speed required by the model when performing computational tasks. Similarly, parameters indicate the model’s complexity and memory footprint, which are crucial for practical deployment. To evaluate computational complexity analysis, the Big-O complexity estimates were used to assess scalability. Moreover, hyperparameter sensitivity analysis was conducted to investigate how variations in model parameters (the number of attention heads, the kernel size, and the number of layers) affect predictive performance. Shapley Additive Explanations (SHAP) analysis was used to interpret feature contributions in the CLSTM model. Finally, the convergence analysis was used to demonstrate training stability through comparative learning curves across multiple influenza seasons.

A box plot was introduced to illustrate the distribution of prediction errors. All data analyses were performed using SPSS Statistics software (version 23; IBM Corporation) and Python (version 3.4.0; Python Software Foundation). All data were checked for completeness and accuracy before analysis.

### Ethical Considerations

The study used deidentified surveillance data from the Chinese NIDRIS in China. The study received formal ethics approval from the research ethics board of Tianjin University (approval number TJUE-2025-222), and the requirement for informed consent was waived. The study did not involve direct human participants research, and no compensation was provided to participants.

## Results

### ILI% Trend From 2013 to 2024

**Figure 2.**
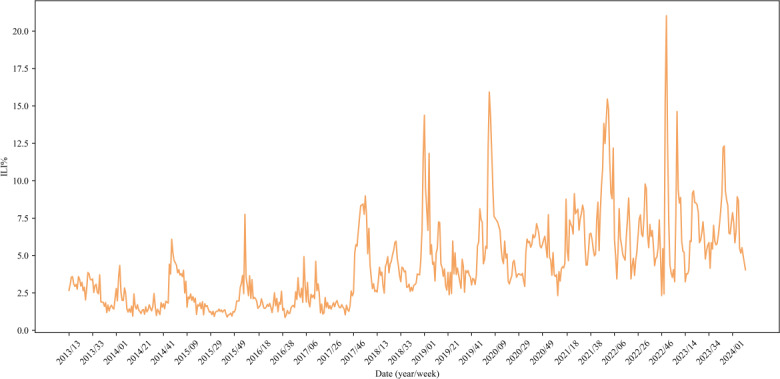
ILI% trend from 2013 to 2024. ILI%: percentage of influenza-like illness.

**Figure 3.**
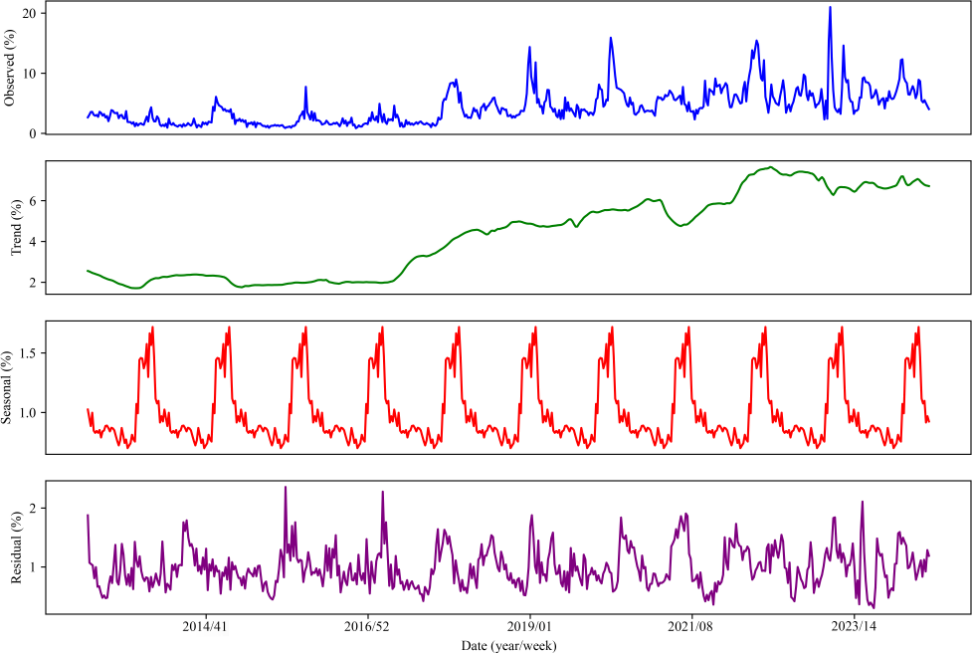
The seasonal and trend decomposition of the percentage influenza-like illness by week from 2013 to 2024.

The ILI% presented a regular seasonal high incidence in China. The average weekly ILI% was 4.36%, respectively. The highest ILI% was in the 50th week in 2022 (21.03%) and the lowest was in the 40th week in 2016 (0.86%). The influenza season is clearly reflected in the period from October to March each year, and there is only 1 influenza peak in the whole influenza cycle (Figure 2). ILI% has a trend of increasing annually (Figure 3). The observed ILI% showed a clear periodic pattern, with the ILI% being higher than the yearly average for the period from October to March (Figure 3).

### Correlation Analysis Between Baidu Index and ILI%

Based on the literature searched on the web, the Baidu search terms in this study that were screened out were all related to influenza. Overall, the study observed a high correlation between the Baidu search terms and the ILI% in all databases. The terms were classified into 4 categories: influenza essential facts, influenza symptoms, influenza treatment, and influenza prevention (Table 1 and Multimedia Appendix 3). The correlation coefficients differed between the influenza epidemic and nonepidemic seasons.

**Table 1.** Correlations between Baidu index and percentage of influenza-like illness in epidemic and nonepidemic seasons at different lags.

Category and terms in Chinese	Terms in English	Cross-correlation coefficient of epidemic season			Cross-correlation coefficient of nonepidemic season		
		Lag 1	Lag 2	Lag 3	Lag 1	Lag 2	Lag 3
Influenza essential facts							
流行性感冒	Influenza	0.692	0.628	0.525	0.421	0.406	0.389
季节性流感	Seasonal influenza	0.587	0.485	0.399	0.364	0.342	0.320
流感传播途径	Influenza transmission route	0.631	0.642	0.589	0.201	0.213	0.279
流感病毒	Influenza virus	0.596	0.531	0.423	0.320	0.320	0.318
流感并发症	Influenza complications	0.546	0.455	0.321	0.484	0.482	0.484
流感流行	Influenza epidemic	0.541	0.518	0.475	0.203	0.197	0.200
上呼吸道感染	Upper respiratory tract infection	0.705	0.620	0.499	0.659	0.641	0.626
小儿感冒	Common cold in children	0.493	0.539	0.564	0.702	0.704	0.686
气管炎	Tracheitis	0.011	0.014	0.071	0.519	0.521	0.530
流感嗜血杆菌	*Haemophilus influenzae*	0.344	0.371	0.380	0.650	0.658	0.667
流感抗原	Influenza antigen	0.315	0.123	0.025	0.531	0.503	0.479
Influenza symptoms							
头痛	Headache	0.181	0.262	0.347	0.551	0.544	0.535
咽痛	Sore throat	0.620	0.468	0.323	0.580	0.536	0.510
发烧	Fever	0.365	0.378	0.388	0.601	0.596	0.591
嗜睡	Drowsiness	0.486	0.468	0.411	0.608	0.597	0.590
咳嗽	Cough	0.514	0.436	0.260	0.036	0.075	0.106
打喷嚏	Sneeze	0.052	0.109	0.196	0.662	0.667	0.681
流鼻涕	Runny nose	0.442	0.453	0.463	0.618	0.615	0.611
流感症状	Influenza symptom	0.406	0.371	0.311	0.654	0.644	0.622
Influenza treatment							
感康	Compound paracetamol and amantadine hydrochloride tablets	0.468	0.281	0.142	0.558	0.507	0.464
感冒清热颗粒	Ganmao Qingre Keli	0.466	0.292	0.136	0.550	0.503	0.464
退烧药	Antipyretics	0.554	0.433	0.355	0.546	0.491	0.447
连花清瘟	Lianhua Qingwen capsule	0.299	0.155	0.065	0.501	0.487	0.475
抗生素	Antibiotic	0.082	0.142	0.201	0.539	0.546	0.532
康泰克	Compound pseudoephedrine HCl^a^-sustained release capsules	0.161	0.030	0.154	0.497	0.537	0.561
清开灵颗粒	Qingkailingkeli	0.210	0.046	0.085	0.484	0.502	0.517
阿莫西林	Amoxicillin	0.669	0.565	0.425	0.656	0.634	0.607
奥司他韦颗粒	Oseltamivir phosphate capsules	0.517	0.496	0.441	0.239	0.394	0.553
抗病毒口服液	Kangbingdukoufuye	0.528	0.338	0.203	0.413	0.354	0.302
流感丸	Liuganwan	0.535	0.387	0.267	0.224	0.199	0.145
菊花茶	Chrysanthemum tea	0.404	0.412	0.400	0.566	0.575	0.582
Influenza prevention							
流感疫苗接种	Influenza vaccine vaccination	0.275	0.241	0.231	0.675	0.672	0.674
流感防控	Influenza prevention and control	0.473	0.429	0.387	0.628	0.624	0.614
姜糖水	Ginger syrup	0.053	0.098	0.244	0.543	0.557	0.566

a HCI: hydrogen chloride.

When analyzing the use of keywords in different time periods, it was found that the focus on keyword categories differed between the influenza epidemic and nonepidemic seasons (Table 1 and Multimedia Appendix 3). Specifically, in the influenza epidemic season, the significant keywords with a cross-correlation coefficient greater than 0.5 mainly concentrated on the category of influenza essential facts. These keywords usually focused on basic information about influenza-related diseases, transmission routes, and other basic information. In contrast, during the nonepidemic season, the most important keywords in the framework mainly focused on the category of influenza treatment. It covered the use of medications, treatment protocols, and other content. This suggests that there are remarkable differences in the public’s concerns during different time periods. In addition, this study observed that the categories of keywords throughout the year mostly overlapped with those used during the epidemic season.

### CLSTM Performance for Prediction

#### Testing the Prediction Results for Different Times in Advance and Various Study Periods

This study used CLSTM for ILI% prediction, with hyperparameter settings detailed in Multimedia Appendix 4. First, the original ILI% was solely input into the framework as the common standard for predicting the ILI% trend. Second, the ILI % was simultaneously input into the framework with a Baidu index with a correlation coefficient above 0.5. The study conducted experiments on 4 different study periods (all-time period, epidemic + nonepidemic period, epidemic period, and nonepidemic period) by adding only ILI% for prediction and ILI% + Baidu index.

**Figure 4.**
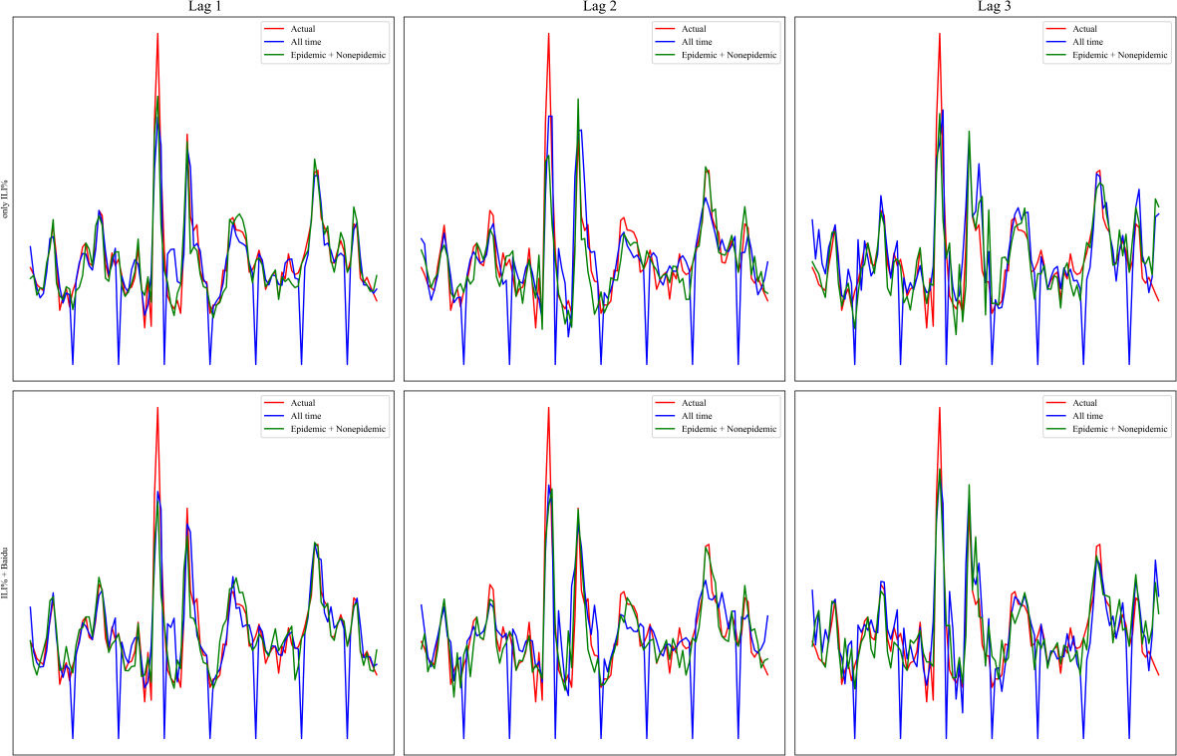
The prediction results for different times in advance and various study periods for all-time period and epidemic + nonepidemic period. There are 3 columns on the graph, lag 1, lag 2, and lag 3, which represent the lag of the dependent variable (***Y***) relative to the independent variable (***X***) of order 1, 2, and 3. There are 2 rows on the graph; the first one is only ILI%, which indicates that only the ILI% indicator is involved in all the inputs and outputs of this framework variable. The second row is ILI% + Baidu, which indicates that the inputs and outputs of this framework variable have both the Baidu index and ILI% involved. The figure was conducted over 2 research periods. First, all-time period (the 11th week of 2022 to the 13th week of 2024). It is an initial time period without a division between epidemic and nonepidemic seasons. Second, epidemic + nonepidemic period (the 11th week of 2022 to the 13th week of 2024). This is represented by dividing the seasons, predicting the results using the respective data, and combining the predictions into a final time period. The 3 lines in the figure represent the predicted values of ILI% for the true value, all-time period, and epidemic + nonepidemic period, respectively. ILI%: percentage of influenza-like illness.

**Figure 5.**
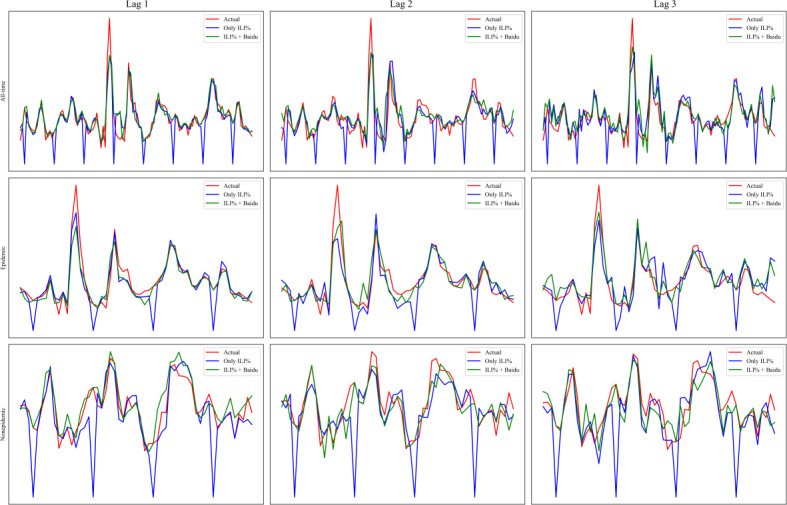
The prediction results for different times in advance and various study periods for only ILI% and ILI% + Baidu. There are 3 columns on the graph, lag 1, lag 2, and lag 3, which represent the lag of the dependent variable (***Y***) relative to the independent variable (***X***) of order 1, 2, and 3. The figure was conducted over 3 research periods. First, all-time period (the 11th week of 2022 to the 13th week of 2024). It is an initial time period without a division between epidemic and nonepidemic seasons. Second, epidemic season (the 11th week of 2022 to the 13th week of 2022, the 40th week of 2022 to the 13th week of 2023, and the 40th week of 2023 to the 13th week of 2024). It indicates the time period of the epidemic season. Third, nonepidemic season (the 14th week of 2022 to the 39th week of 2022, the 14th week of 2023 to the 39th week of 2023). It indicates the time period of the nonepidemic season. There are 3 rows on the chart, each representing 3 time periods. The 3 lines on each graph are the predicted values of ILI% at the true value, the case where only ILI% is the variable, and the case where ILI% + Baidu is the variable, respectively. ILI%: percentage of influenza-like illness.

It was found that the prediction of ILI% after dividing the epidemic and nonepidemic seasons was better than that of the all-time period (Figure 4 and Table 2). In particular, the effects of 1-week and 2-week lag predictions were more stable (lag 1 of ILI% in period 2: MAPE=10.730%, MSE=0.884, MAE=0.649, RMSE=0.940, and *R*^2^=0.877; lag 1 of ILI% + Baidu index in period 2: MAPE=11.824%, MSE=1.243, MAE=0.723, RMSE=1.115, and *R*^2^=0.827). On the prediction effect of ILI% 3-week lag, using only ILI% is better than ILI% + Baidu index (lag 3 of ILI% in period 2: MAPE=18.458%, MSE=2.623, MAE=1.053, RMSE=1.620, and *R*^2^=0.635; lag 3 of ILI% + Baidu index in period 2: MAPE=18.260%, MSE=1.996, MAE=1.014, RMSE=1.413, and *R*^2^=0.722; Table 2). In addition, it was found that the ILI% + Baidu index predicts better than only the ILI% regardless of the time period and lag time of the study (Figure 5 and Table 2). The conclusions were general in nature and demonstrated stable model performance.

**Table 2.** Prediction effects for different study periods and for different combinations of inputs^a^.

	ILI%^b^					ILI% + Baidu index				
	MAPE (%)^c^	MSE^d^	MAE^e^	RMSE^f^	*R* ^2^	MAPE (%)	MSE	MAE	RMSE	*R* ^2^
Period 1: all-time										
Lag 1	12.784	1.513	0.744	1.230	0.786	13.464	1.377	0.762	1.173	0.806
Lag 2	15.913	1.800	0.924	1.342	0.746	15.737	1.718	0.917	1.311	0.758
Lag 3	15.287	1.815	0.881	1.347	0.744	17.544	1.934	0.978	1.391	0.727
Period 2: epidemic + nonepidemic										
Lag 1	10.730	0.884	0.649	0.940	0.877	11.824	1.243	0.723	1.115	0.827
Lag 2	13.257	1.681	0.858	1.297	0.766	13.235	1.522	0.816	1.234	0.788
Lag 3	18.458	2.623	1.053	1.620	0.635	18.260	1.996	1.014	1.413	0.722
Period 3: epidemic										
Lag 1	12.089	2.001	0.831	1.415	0.818	11.966	1.603	0.782	1.266	0.854
Lag 2	14.436	2.189	0.955	1.480	0.801	14.707	1.864	0.934	1.365	0.831
Lag 3	20.637	3.286	1.203	1.813	0.702	22.368	2.865	1.232	1.693	0.740
Period 4: nonepidemic										
Lag 1	9.218	0.478	0.548	0.691	0.813	8.480	0.378	0.479	0.615	0.852
Lag 2	10.189	0.607	0.612	0.779	0.763	9.753	0.574	0.555	0.758	0.775
Lag 3	10.863	0.733	0.638	0.856	0.713	11.634	0.715	0.670	0.846	0.720

a The study was conducted over 4 research periods. Period 1: all-time period (the 11th week of 2022 to the 13th week of 2024). It is an initial time period without a division between epidemic and nonepidemic seasons. Period 2: epidemic + nonepidemic period (the 11th week of 2022 to the 13th week of 2024). This is represented by dividing the seasons, predicting the results using the respective data, and combining the predictions into a final time period. Period 3: epidemic period (the 11th week of 2022 to the 13th week of 2022, the 40th week of 2022 to the 13th week of 2023, and the 40th week of 2023 to the 13th week of 2024). It indicates the time period of the epidemic season. Period 4: nonepidemic period (the 14th week of 2022 to the 39th week of 2022, and the 14th week of 2023 to the 39th week of 2023). It indicates the time period of the nonepidemic season.

b ILI%: percentage of influenza-like illness.

c MAPE: mean absolute percentage error.

d MSE: mean square error.

e MAE: mean absolute error.

f RMSE: root-mean-square error.

**Figure 6.**
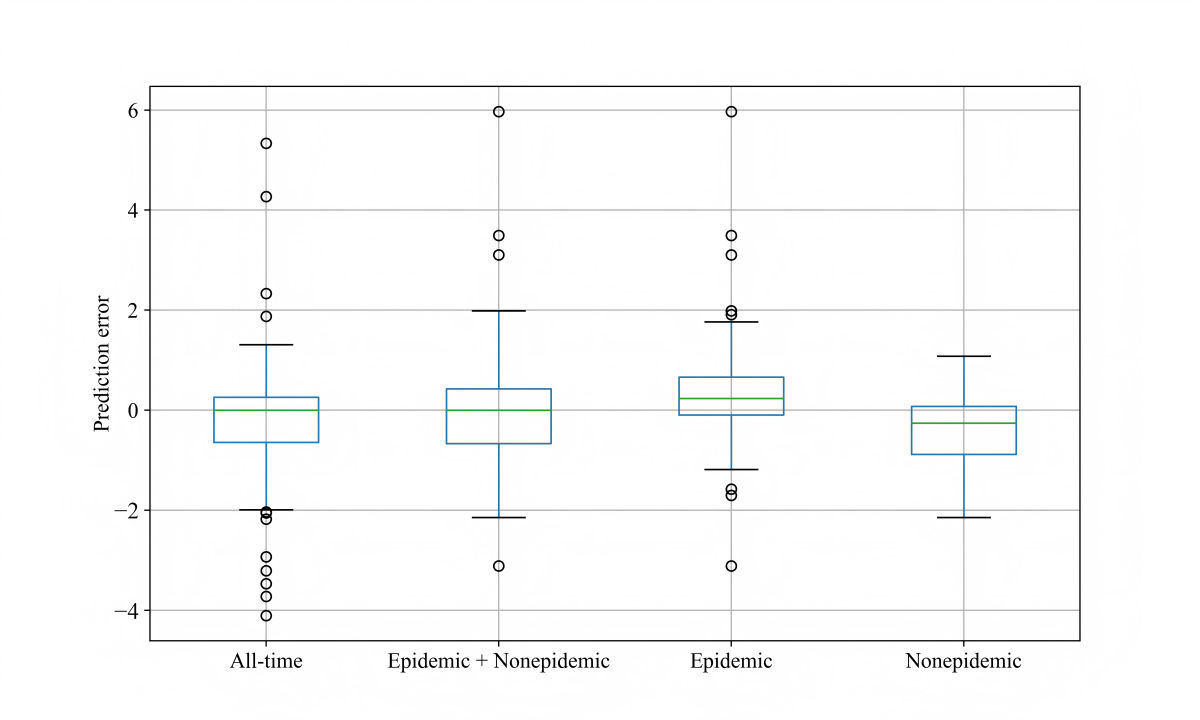
Convolutional long short-term memory prediction error distribution in 1-week-ahead ILI% predictions using ILI% + Baidu index. Four different periods: all-time, epidemic + nonepidemic, epidemic, and nonepidemic. Each period is represented by a box plot, where the green horizontal line indicates the median of the data, the blue box represents the interquartile range from the first quartile (**Q1**) to the third quartile (**Q3**), and any data points outside the whiskers are marked with circles, indicating potential outliers. ILI%: percentage of influenza-like illness.

CLSTM demonstrates robust reliability for 1-week-ahead ILI% predictions using ILI% + Baidu index data in 4 periods (all-time, epidemic + nonepidemic, epidemic, and nonepidemic periods). The overall prediction errors exhibit a median near zero with reasonable distribution ranges (Figure 6). Notably, the model maintains stable performance even during the most challenging epidemic period, while achieving tighter error distributions in the nonepidemic period. These results confirm the practical reliability of our integrated model for real-world influenza surveillance applications.

#### Explainability of Model Predictions

To validate the prediction reliability of the model, SHAP analysis was used to assess feature importance based on ILI% + Baidu index data in epidemic + nonepidemic period. The results demonstrate that both the Baidu index (SHAP value=2.487) and the ILI% (SHAP value=3.172) contribute significantly to the predictive outcomes (Multimedia Appendix 5). While ILI% exhibits slightly higher importance, the Baidu index provides complementary search behavior data that effectively compensate for the limitations of traditional surveillance indicators, particularly in capturing early warning signals. This finding confirms the value of integrating web-based search data with conventional epidemiological data, offering more comprehensive support for influenza forecasting.

#### Hyperparameter Sensitivity Analysis

This study conducted a hyperparameter sensitivity analysis for 1-week-ahead ILI% predictions using ILI% + Baidu index data in epidemic + nonepidemic period to evaluate 3 key parameters (Table 3): the number of attention heads (Num_head) in transformer, the kernel size (Kernel) in convolutional neural networks, and the number of layers (Num_layer) in LSTM. While holding 2 hyperparameters constant, it systematically varied the third hyperparameter and evaluated model performance. This procedure yielded a total of 7 comparative experiments for hyperparameter sensitivity analysis. The experimental results demonstrated that the optimal configuration (Num_head=8, Kernel=9, and Num_layer=2) achieved superior performance across all evaluation metrics. Based on these findings, this study implemented this optimal hyperparameter configuration in the final model to ensure robust predictive performance.

**Table 3.** Hyperparameter sensitivity analysis of convolutional long short-term memory.

Hyperparameter			MAPE^d^ (%)	MSE^e^	MAE^f^	RMSE^g^	*R* ^2^
Num_head^a^	Kernel^b^	Num_layer^c^					
4	9	2	11.831	1.518	0.757	1.232	0.826
8	9	2	11.824	1.243	0.723	1.115	0.827
16	9	2	12.554	1.692	0.833	1.301	0.801
8	1	2	14.695	2.408	0.969	1.552	0.781
8	3	2	15.570	2.603	1.103	1.613	0.764
8	9	2	11.824	1.243	0.723	1.115	0.827
8	9	2	11.824	1.243	0.723	1.115	0.827
8	9	3	11.934	1.580	0.769	1.257	0.817
8	9	4	17.100	2.742	1.137	1.656	0.751

a Num_head: the number of attention heads in transformer.

b Kernel: the kernel size in convolutional neural networks.

c Num_layer: the number of layers in long short-term memory.

d MAPE: mean absolute percentage error.

e MSE: mean square error.

f MAE: mean absolute error.

g RMSE: root-mean-square error.

### Comparisons With Other Methods

#### Models’ Performance for Prediction

**Figure 7.**
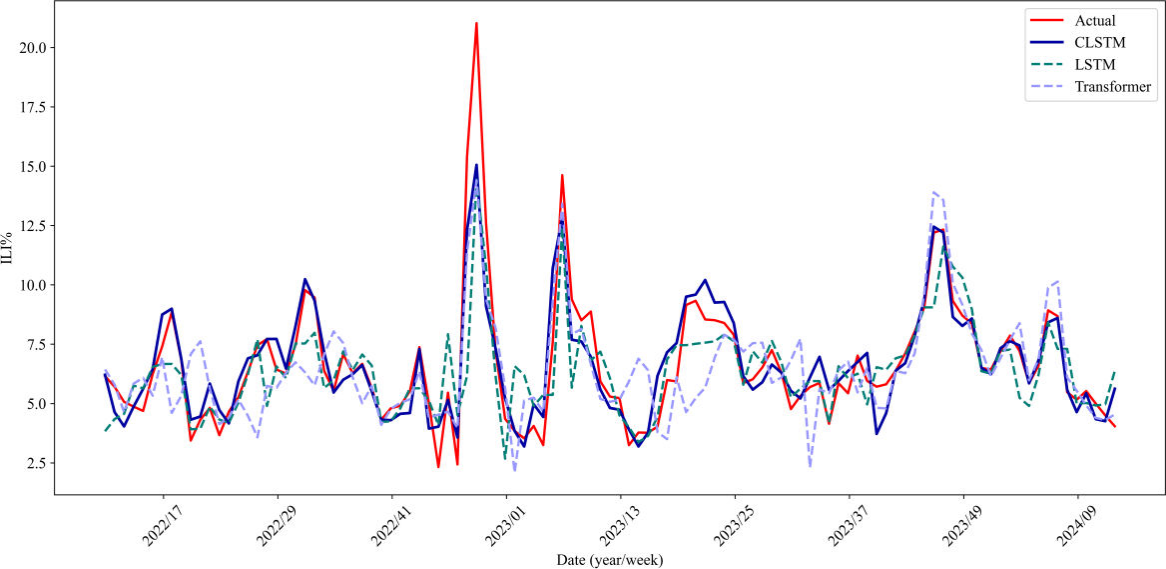
Comparison of 3 model prediction performance for 1-week-ahead ILI% predictions using ILI% + Baidu index data in epidemic + nonepidemic period. CLSTM: convolutional long short-term memory; ILI%: percentage of influenza-like illness; LSTM: long short-term memory.

The comparative performance evaluation of CLSTM against traditional LSTM and transformer architectures was conducted for 1-week-ahead ILI% predictions using ILI% + Baidu index data in epidemic + nonepidemic period (Table 4 and Figure 7). Quantitative analysis revealed that CLSTM consistently outperformed baseline models across all evaluation metrics, achieving superior performance in prediction accuracy (MSE: 1.243 vs 2.230/2.009; MAE: 0.723 vs 0.654/0.705; and RMSE: 1.115 vs 1.493/1.417), stability (MAPE: 11.824% vs 14.458%/13.561%), and explanatory power (*R*²: 0.827 vs 0.744/0.803). The performance advantage was particularly pronounced during interseasonal transitions between epidemic and nonepidemic periods, demonstrating CLSTM’s enhanced capability in modeling complex nonlinear relationships and long-term temporal dependencies inherent in syndromic surveillance data. These results substantiate the effectiveness of our proposed architecture for ILI prediction tasks under real-world surveillance scenarios.

**Table 4.** Evaluation metrics of 3 model prediction performance^a^.

Model	MAPE^b^ (%)	MSE^c^	MAE^d^	RMSE^e^	*R* ^2^
LSTM^f^	14.458	2.230	0.654	1.493	0.744
Transformer	13.561	2.009	0.705	1.417	0.803
CLSTM^g^	11.824	1.243	0.723	1.115	0.827

a The comparative performance evaluation of convolutional long short-term memory against traditional long short-term memory and transformer architectures was conducted for 1-week-ahead percentage of influenza-like illness predictions using the percentage of influenza-like illness + Baidu index data in epidemic + nonepidemic period.

b MAPE: mean absolute percentage error.

c MSE: mean square error.

d MAE: mean absolute error.

e RMSE: root-mean-square error.

f LSTM: long short-term memory.

g CLSTM: convolutional long short-term memory.

#### Model Computational Efficiency and Complexity Analysis

##### Model Computational Efficiency

For model computational efficiency, the study used FLOPs and the number of parameters as evaluation metrics (Table 5). FLOPs can be used to measure computational efficiency because it directly reflects the amount of computational resources and processing speed required by the model when performing computational tasks. Similarly, parameters indicate the model’s complexity and memory footprint, which are crucial for practical deployment. The results show that while the CLSTM model has a higher FLOPs value (0.40G) than the LSTM (0.08G), it is still lower than the transformer’s FLOPs (0.48G). Additionally, the CLSTM model has fewer parameters (2.88M) than the transformer (3.44M) but more than the LSTM (0.47M). This indicates that CLSTM achieves a good balance between computational complexity, memory usage, and performance.

**Table 5.** Model computational complexity analysis.

Model	FLOPs^a^ (GB)	Parameters (MB)	Big-O
LSTM^b^	0.08	0.47	O (T^c^·d²^d^)
Transformer	0.48	3.44	O (N·T²·d)
CLSTM^e^	0.40	2.88	O (E^f^ ·n·m·k·(*f*+h^g^))

a FLOPs: floating-point operations per second.

b LSTM: long short-term memory.

c T: input sequence length.

d d: input feature dimension.

e CLSTM: convolutional long short-term memory.

f E: epoch.

g h: hidden state dimension.

##### Model Computational Complexity Analysis

The computational complexity analysis reveals the performance differences among LSTM, transformer, and CLSTM (Table 5). First, LSTM’s O(T·d²) limits its scalability with high-dimensional data; second, transformer’s O(N·T²·d) incurs higher computational costs due to the number of attention heads. In contrast, CLSTM’s O (E·n·m·k·(*f*+h)), although influenced by multiple factors, can achieve greater efficiency through optimization of batch size and filter dimensions. It can make it more scalable for tasks involving complex features and long sequences. The derivation process for the Big-O complexity estimation is shown in Multimedia Appendix 6.

### Comparing Training Stability Across Different Influenza Periods

**Figure 8.**
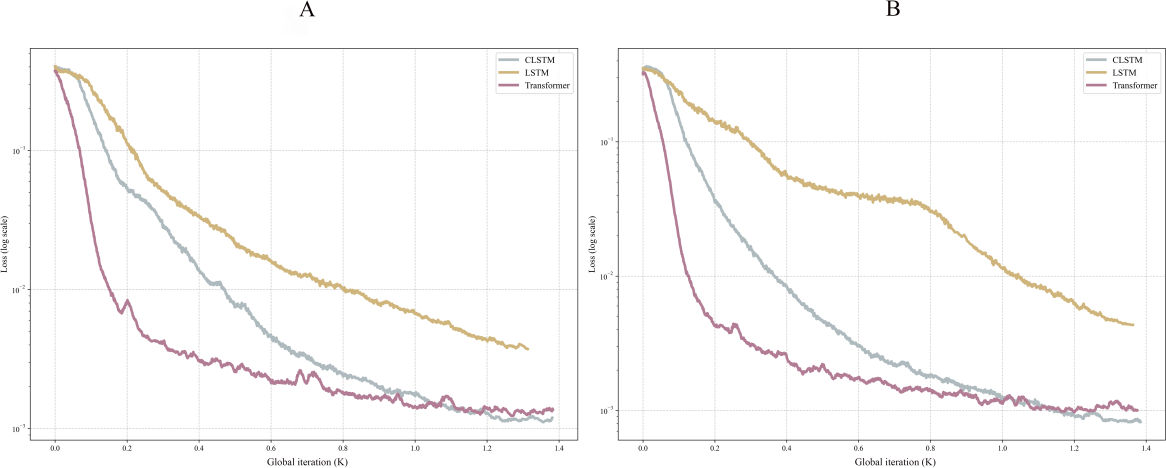
Loss functions of 3 models (CLSTM, transformer, and LSTM). (A) Epidemic period. (B) Nonepidemic period. CLSTM: convolutional long short-term memory; LSTM: long short-term memory.

To compare the training stability of models across epidemic and nonepidemic periods, this study presents the loss functions of 3 models (CLSTM, transformer, and LSTM) (Figure 8A and B). Comparative analysis revealed consistent performance rankings: transformer achieved optimal convergence, LSTM exhibited the weakest performance, and CLSTM maintained robust intermediate performance. CLSTM demonstrated remarkable stability despite seasonal variations in data distribution, highlighting superior robustness and cross-distribution adaptability. All-time period convergence analysis (Multimedia Appendix 7) yielded consistent results with seasonal evaluations.

### External Validation

To further validate the generalizability of the CLSTM model, the study conducted additional experiments using ILI% from Tianjin and corresponding Baidu index data. The model demonstrated robust performance for 1-week-ahead ILI% predictions using ILI% + Baidu index data in epidemic + nonepidemic period (Table 6), achieving an *R*² of 0.755 >0.7, MAPE of 17.832% <20%, and low error metrics (RMSE=1.716, MSE=2.943, and MAE=1.216). These results indicate the model’s strong predictive capability on external datasets. Comparative analysis revealed the CLSTM model’s superior performance over LSTM and transformer. It confirmed the model’s enhanced ability to capture spatiotemporal patterns in ILI transmission.

**Table 6.** Evaluation metrics of 3 model prediction performance in external validation^a^.

Model	MAPE^b^ (%)	MSE^c^	MAE^d^	RMSE^e^	*R* ^2^
LSTM^f^	26.458	4.065	2.988	2.016	0.700
Transformer	21.887	3.111	2.001	1.764	0.743
CLSTM^g^	17.832	2.943	1.216	1.716	0.755

a The comparative performance evaluation of convolutional long short-term memory against traditional long short-term memory and transformer architectures was conducted for 1-week-ahead percentage of influenza-like illness predictions using the percentage of influenza-like illness + Baidu index data in epidemic + nonepidemic period.

b MAPE: mean absolute percentage error.

c MSE: mean square error.

d MAE: mean absolute error.

e RMSE: root-mean-square error.

f LSTM: long short-term memory.

g CLSTM: convolutional long short-term memory.

## Discussion

### Principal Findings

Although current models of transmission epidemiology largely rely on surveillance data and mathematical models, there have been many studies exploring the potential of alternative data sources and machine learning techniques. This study integrates Baidu index data with traditional surveillance data to propose a deep learning framework that uses multifeatured time series for different influenza epidemic states. By considering the impacts of both epidemic and nonepidemic seasons, the framework enhances the accuracy of influenza predictions. Analysis revealed that the weekly search volumes for 4 types of Baidu search terms related to influenza—essential facts, symptoms, treatment, and prevention—were strongly correlated with ILI%. By distinguishing between epidemic and nonepidemic seasons, this study found that these search terms had different focuses during each period. Based on these findings, we developed a CLSTM deep learning framework using multifeatured time series from multiple data sources. The study was conducted over 4 research periods: all time periods, the combined epidemic and nonepidemic periods, the epidemic period, and the nonepidemic period. The framework can predict influenza trends up to 2 weeks in advance. This research investigates the application of combining public opinion data with traditional surveillance systems to predict influenza epidemics. It provides a valuable reference not only for northern China but also for advancements in modern surveillance methods.

Dividing the study period into epidemic and nonepidemic seasons can more accurately capture the seasonal characteristics of the public’s search behavior for influenza information (Baidu index). The ILI% was used to predict trends in influenza virus incidence [32]. An increase in the ILI% usually signals the peak of an influenza pandemic, and health care providers and public health departments can take timely action based on the change in the ILI% to intensify their influenza prevention and treatment efforts. Nevertheless, influenza risks vary during different seasons. Therefore, dividing the study period into epidemic and nonepidemic seasons better captures seasonal variations in public concern and search behavior, which are crucial for predicting influenza trends. During the influenza epidemic season, members of the public, with heightened crisis awareness and perceived increased risk of contracting influenza, proactively obtain relevant information for self-assessment and prevention. Specifically, they pay attention to the symptoms of influenza for self-diagnosis, learn about transmission routes to prevent infection, and seek preventive measures to protect themselves. These behaviors led to a significant increase in Baidu search terms related to influenza essential facts and prevention, which in turn led to an increase in the Baidu index. In addition, extensive media coverage of influenza cases and outbreak developments also increased public attention. Public health departments usually promote preventive measures such as vaccination and hand hygiene during the epidemic season, which further directs the public to search for related information. In contrast, during the nonepidemic season, the public is relatively less aware of the crisis and less worried about the risk of contracting influenza. In this case, they mainly focus on information related to influenza treatment. Therefore, Baidu search terms focus more on recognizing influenza symptoms, choosing treatments, and effective medications. Media reports and other health information received relatively less attention, while public interest in vaccination and preventive measures declined. Overall, public attention during the nonepidemic season shifted more toward the influenza treatment.

The inclusion of Baidu index is effective for ILI% prediction. Using separate Baidu search terms for epidemic and nonepidemic seasons will improve prediction performance. The search engine data, as a leading signal of influenza trends, can sense the spreading trend of influenza earlier than the ILI% [27]. Baidu index is a globally applicable big data tool, widely used in Baidu-dominated regions (eg, parts of Asia and South America). By the second quarter of 2025, Baidu will have a wide range of active users globally. Baidu operates similarly to Google Trends and other international search indices, making the model adaptable to local platforms (eg, Naver in Korea) without algorithmic modifications. Currently, Baidu index has been used in deep learning–based prediction of influenza on several occasions [31,33,34]. Search terms filtered from Baidu index are often divided into 4 categories [31,34,35]: basic understanding of influenza, symptoms, treatment and medicine, and prevention. It is generally consistent with this study’s categorization of influenza-related terms.

At the same time, it is necessary to study influenza prediction using deep learning algorithms. Yang et al [34] used data from northern and southern China and integrated a model based on gated recurrent units and multiple attention mechanisms to achieve approximately 2 weeks’ prediction, with an *R*² value exceeding 0.77. Jung et al [36] used a self-attention mechanism-based deep learning model to predict influenza trends, demonstrating significant effectiveness. Additionally, studies [31,33] combined ILI case, virological surveillance, climate, demographic, and search engine data. They applied LSTM models to predict ILI%, achieving *R*² values from 0.67 to 0.9. Athanasiou et al [35] used weekly ILI monitoring data and multisource data, including Twitter and weather conditions, to predict ILI% in Greece with an LSTM model. In this study, using only Baidu index and traditional monitoring data, the CLSTM framework predicted ILI% in 1-2 weeks. The framework’s *R*^2^ value exceeded 0.82, outperforming most studies using multisource data. Comparative analysis demonstrates that CLSTM achieves better performance to classical machine learning models in terms of computational efficiency, complexity, extrapolation capability, and stability.

It was found that the current papers rarely achieve more than 3 weeks of accurate prediction, which is deemed ineffective [31,35]. The Baidu index reflects public search behavior for influenza-related information. It typically increases 1-2 weeks before an outbreak and weakens afterward, reducing predictive effectiveness. Traditional ILI% surveillance data are more reliable for longer-term predictions and provide comprehensive information.

Most influenza prediction studies do not divide epidemic and nonepidemic seasons. This study found that using specific Baidu search terms for each period significantly improves prediction performance and reliability during single epidemic or nonepidemic seasons. This approach allows the framework to capture season-specific patterns more accurately, providing reliable and detailed prediction results.

### Limitations and Future Works

However, this study had a few limitations. First, this study did not separately consider the effect of the COVID-19 pandemic on ILI% but allowed the framework to learn from existing trends. Second, computational challenges (eg, real-time latency) must be addressed through optimization and cloud solutions.

To enhance robustness, future work should explore the following: first, hybrid artificial intelligence strategies (eg, reinforcement learning) for dynamic parameter tuning; second, multisource data integration (social media, electronic health records, and climate variables) to improve sensitivity and reduce confounders; and third, federated learning for privacy-preserving cross-region collaboration, despite computational inequities.

### Conclusions

In summary, this study demonstrates significant potential for influenza prediction by integrating Baidu index with traditional surveillance data and specific keywords (for both epidemic and nonepidemic seasons). The CLSTM framework performed exceptionally well, effectively improving prediction accuracy through the use of seasonal Baidu search terms, thereby offering a novel perspective for public health early warning systems. Although Baidu’s search engine is widely used internationally, its data are subject to demographic, geographical, and media-driven biases, necessitating population-weighting adjustments and integration with traditional surveillance data to mitigate prediction distortions. Future efforts will focus on further optimizing the model to achieve more timely and accurate predictions: on one hand, leveraging transfer learning to extend this framework to other disease predictions, and on the other hand, using multiobjective optimization to balance accuracy and interpretability. It will ultimately enhance public health response capabilities.

## Supplementary material

10.2196/71786 Multimedia Appendix 1 Mathematical formulation of the convolutional long short-term memory framework.

10.2196/71786 Multimedia Appendix 2 Model evaluation.

10.2196/71786 Multimedia Appendix 3 Correlations between Baidu index and percentage of influenza-like illness in all-time period at different lags.

10.2196/71786 Multimedia Appendix 4 Implementation details of the convolutional long short-term memory framework for the percentage of influenza-like illness.

10.2196/71786 Multimedia Appendix 5 Shapley Additive Explanations analysis.

10.2196/71786 Multimedia Appendix 6 Derivation process and results of Big-O complexity estimation.

10.2196/71786 Multimedia Appendix 7 Convergence analysis for all-time period.

## Abbreviations

CLSTM: convolutional long short-term memory
FLOP: floating-point operation per second
ILI: influenza-like illness
ILI%: percentage of influenza-like illness
LSTM: long short-term memory
MAE: mean absolute error
MAPE: mean absolute percentage error
MSE: mean square error
NIDRIS: National Notifiable Infectious Disease Reporting System
RMSE: root-mean-square error
SHAP: Shapley Additive Explanations
