# Supplementary material for: A Deep Learning Framework for Using Search Engine Data to Predict Influenza-Like Illness and Distinguish Epidemic and Nonepidemic Seasons: Multifeature Time Series Analysis
Source: J Med Internet Res. 2025 Aug 11;27:e71786. doi: 10.2196/71786 (PMC12338964; doi:10.2196/71786)
Supplement: Multimedia Appendix 1 [file jmir-v27-e71786-s001.docx]

**Multimedia Appendix 1 Mathematical Formulation of the CLSTM Framework**

CLSTM uses a classic encoder-decoder structure to build its algorithm framework. The encoder consists of a backbone and a fusion part.

**1 Encoder**

**1.1 Backbone - Variational mode decomposition**

When modeling high-frequency data, using raw unprocessed inputs often reduces model performance. It may even produce worse results than low-frequency data. This occurs because high-frequency data usually contains substantial noise. Such interference severely affects feature extraction. This leads to biased learning outcomes. Two mainstream signal processing methods exist: wavelet decomposition and variational mode decomposition (VMD). Traditional wavelet decomposition requires manual setting of key parameters. These include basis functions and decomposition levels. It also lacks adaptability for different high-frequency data types [1]. In comparison, VMD can adaptively determine optimal decomposition modes. Theoretically, it offers better noise suppression. VMD shows particular advantages for non-stationary high-frequency time-series data. Its unique decomposition mechanism gives it superior performance.

**1.2 Backbone -Transformer**

The Transformer’s self-attention mechanism can analyze entire time series simultaneously. It effectively captures long-range dependencies. Compared to RNN’s sequential processing, Transformer more accurately identifies cross-timestep patterns. It demonstrates superior performance in long-term time series forecasting [2]. The Transformer architecture primarily consists of these key components:

①Self-attention mechanism. he self-attention mechanism is the core component of Transformer. It models global dependencies by calculating interaction weights between all positions in the sequence. In the computational framework of self-attention, three core matrices serve different functional roles: The query matrix *Q* represents request vectors for current information needs; The key matrix *K* stores feature vectors to be matched; The value matrix *V* contains actual feature information. Here, *d_k_* represents the dimension parameter of key vectors. This parameter serves a normalization role in computing attention weights, ensuring numerical stability of dot product results. The self-attention formula is expressed as:

Where *Q* is the query matrix, *K* is the key matrix, *V* is the value matrix, and *dₖ* is the dimension parameter of key vectors.

②Multi-head attention mechanism. The multi-head attention mechanism adopts a spatial decomposition strategy. It projects input features into several independent feature subspaces. Within each subspace, the system performs self-attention operations in parallel to obtain local feature interaction patterns. By integrating output features from all subspaces, the model can simultaneously model various types of dependency relationships. The multi-head attention mechanism formula is expressed as:

Where *Q* is the query matrix, *K* is the key matrix, *V* is the value matrix, *W_i_^Q^*, *W_i_^K^*, *W_i_^V^* and *W^o^* are trainable weight matrices.

③Positional encoding. The Transformer’s core architecture addresses sequence order awareness through positional encoding. The positional encoding formula for even positions is expressed as:

The positional encoding formula for odd positions is expressed as:

Where *pos* represents position, *i* is the dimension index, and *d_model_* is the model’s dimension.

④Feedforward neural network. The Transformer employs a feedforward neural network after each attention layer. This structure processes features at each position independently. It enhances the model’s nonlinear expressive capability while maintaining position independence. The feedforward neural network formula is expressed as:

Where *x* is the input, *W_1_* and *W_2_* are weight matrices, *b_1_* and *b_2_* are bias parameters.

⑤Residual connection and layer normalization. To improve training stability in deep neural networks, the model incorporates residual connections and layer normalization mechanisms in each sublayer. This includes both self-attention modules and feedforward networks. The final output y is expressed as:

Where *x* is the input and $Sublayer\left(x\right)$ is the sublayer’s output.

**1.3 Backbone -Convolutional long short-term memory network**

This study chooses to incorporate CNN when building the LSTM model. This creates a CNN-LSTM model [3] that combines the analytical strengths of both approaches.

The CNN-LSTM model generally includes four structures:

①Input layer. The input consists of time-series data, including time steps and feature vectors. To ensure the model's effectiveness, raw data must undergo preprocessing, and the processed data is then constructed into a feature matrix to serve as the input for subsequent layers.

②CNN layer. Composed of convolutional layers, pooling layers, and others, the convolutional layers extract data features, while the pooling layers filter information. Finally, a fully connected layer transforms the data into a form suitable for further processing.

③LSTM layer. Consisting of multiple LSTM units, including a forget gate, input gate, and output gate, this layer processes the information from the CNN layer.

④Output layer. After feature extraction and information processing by the CNN and LSTM layers, the results are output through a fully connected layer.

**1.4 Fusion -** **Gated attention fusion**

GAF module is designed for integrating diverse feature information. By leveraging a gated attention mechanism, GAF effectively combines data from different sources. Particularly in time-series forecasting tasks, it excels at capturing complex dependencies among various features.

①Computing feature correlations. The gated attention mechanism, as the core module of GAF, calculates the correlations between different features to capture their interdependencies. By applying linear transformations to the input features, it generates Query (*Q*), Key (*K*), and Value (*V*) matrices, expressed as:

Where *Q* is the query matrix, *K* is the key matrix, *V* is the value matrix, *W_q_*, *W_k_* and *W_v_* are trainable weight matrices, *M* is the input feature matrix.

②Allocating attention weights. After obtaining Q and K, this step computes their similarity and applies softmax normalization to generate attention weights. These weights reflect the contribution degree of each feature to the final output.

Where *Q* is the query matrix, *K* is the key matrix, *V* is the value matrix, and *dₖ* is the dimension parameter of key vectors.

③Generating gating signals. The gating signals are used to further refine the attention weights by selectively focusing on key features. This mechanism enables the model to adaptively prioritize the most influential features for prediction. The gating signal is generated as follows:

Where *W_g_* and *b_g_* are learnable weights and biases, $\sigma$ is the sigmoid function, and [*Q,K,V*] is the concatenation of the query matrix (Query), key matrix (Key), and value matrix (Value).

④Calculate final gated weights. The gating signal adjusts initial attention weights to generate final gated attention weights. The formula is:

Where ⊙ denotes element-wise multiplication.

⑤Weighted sum of values. The attention weights from the gated attention mechanism perform weighted summation on values. This process integrates contributions from different features. It generates a comprehensive feature representation where more important features have higher weights. The formula for weighted summation using attention weights is:

Where $\alpha_{i}$ is the attention weight for the *i*-th feature and *V_i_* is the value of the *i*-th feature.

⑥Perform global pooling. After feature fusion, GAF further extracts global feature information through global pooling. Global pooling reduces feature dimensions while retaining important global information. This provides a more compact feature representation for the decoder. The global pooling formula is:

Where max denotes max pooling.

⑦Generate decoder input. The combined feature *M_y_* is fed into the decoder for further time-series prediction tasks. Through these key components, GAF effectively integrates features from different sources. It captures complex dependencies between features and generates final predictions. This improves time-series forecasting performance. The formulas for combined feature *M_y_* and final predicted results are:

Where *M_y_* is the combined feature fed into the decoder. Predicted ILPs are the model’s final prediction results.

**2 Decoder**

The decoder section consists of CNN-LSTM and MLP algorithms. The CNN-LSTM model was introduced earlier and won’t be repeated here. MLP is a typical feedforward neural network architecture. Its topology includes three basic components: input layer, hidden layer, and output layer [4]. The basic MLP structure is as follows:

①Input layer. It primarily handles data preprocessing. For time-series forecasting, this layer receives historical observations. These require standardization or normalization to eliminate unit differences affecting model performance.

②Hidden layer. The hidden layer consists of multiple nonlinear computing units. Its core computation involves two key steps. First, linear weighting of previous layer outputs. Then nonlinear transformation through activation functions like ReLU or tanh. This structure enables the network to fit complex functional relationships. Deep stacking increases model capacity but significantly raises computational complexity.

③Output layer. It generates predicted values. Single time-step prediction requires only one output node. Multi time-step prediction requires output dimensions matching the forecast length. This ensures model output aligns with task requirements.

The MLP information transfer mechanism can be decomposed into layer-by-layer computation. The formulas are as follows:

Input layer to hidden layer computation:

Where *h_j_* is the output of the *j*-th hidden layer neuron. $x_{i}$ is the raw input feature signal from the *i*-th input node. *w_ij_* is the connection weight from input to hidden layer. *b_j_* is the bias parameter for the *j*-th hidden neuron. $\sigma$is the sigmoid function.

Hidden layer to output layer computation:

Where $y_{k}$ is the output of the *k*-th output node. *w_jk_* is the connection weight from hidden to output layer. *b_k_* is the bias parameter for the *k*-th output node. $\sigma$ is the sigmoid function.

**References**

1. Zhang X, Li H, Rong W. Reliable Denoising Strategy to Enhance the Accuracy of Arrival Time Picking of Noisy Microseismic Recordings. Sensors. 2023 Nov 26;23(23):9421.

2. Vaswani A, Shazeer N, Parmar N, Uszkoreit J, Jones L, Gomez AN, et al. Attention Is All You Need. arXiv; 2023, http://arxiv.org/abs/1706.03762.

3. Li G, Li Y, Han G, Jiang C, Geng M, Guo N, et al. Forecasting and analyzing influenza activity in Hebei Province, China, using a CNN-LSTM hybrid model. BMC Public Health. 2024 Aug 12;24(1):2171.

4. Shi Y, Zhang G, Ma C, Xu J, Xu K, Zhang W, et al. Machine learning algorithms to predict intraoperative hemorrhage in surgical patients: a modeling study of real-world data in Shanghai, China. BMC Med Inform Decis Mak. 2023 Aug 10;23(1):156.
