# Supplementary material for: A Deep Learning Framework for Using Search Engine Data to Predict Influenza-Like Illness and Distinguish Epidemic and Nonepidemic Seasons: Multifeature Time Series Analysis
Source: J Med Internet Res. 2025 Aug 11;27:e71786. doi: 10.2196/71786 (PMC12338964; doi:10.2196/71786)
Supplement: Multimedia Appendix 2 [file jmir-v27-e71786-s002.docx]

**Multimedia Appendix 2 Model evaluation**

This study uses three types of indicators to evaluate the performance of the prediction model, including model explanatory power metric, accuracy metrics, and stability metric.

Explanatory power metric mainly uses the coefficient of determination (R-squared, R^2^). R^2^ is a statistical measure that represents the proportion of the variance for a dependent variable that’s explained by an independent variable or variables in a regression model. It serves as an indicator of how well the model’s predictions match the actual observed data. R^2^ is calculated by comparing the total variation in the dependent variable to the variation that is unexplained by the model. The R² score ranges from 0 to 1, with values closer to 1 indicating better model fit and stronger explanatory power. Generally, an R² above 0.7 is considered to have high explanatory capability.

$$\begin{aligned} \#\left( 1 \right) \end{aligned}$$

Accuracy metrics include mean square error (MSE), root mean square error (RMSE), and mean absolute error (MAE). The range of these metrics is from 0 to positive infinity, with values closer to 0 indicating smaller prediction errors and higher model accuracy.

MSE is a widely used metric for quantifying the disparity between a model’s predicted values and the actual observed values, serving as an indicator of how well the model fits the provided dataset. MSE is calculated by finding the mean of the squared diferences between the predicted values and the actual observed values.

$$\begin{aligned} \#\left( 2 \right) \end{aligned}$$

RMSE is another commonly employed metric to assess the dissimilarity between a model’s predicted values and the actual observed values, providing insight into the model’s fit to the given data. RMSE is determined by computing the mean of the squared differences between predicted values and actual observations, followed by taking the square root of the result.

$$\begin{aligned} \#\left( 3 \right) \end{aligned}$$

MAE is a frequently used measure for assessing the divergence between a model’s predicted and actual observations, indicating the model’s fit to the provided data. MAE is derived by calculating the mean of the absolute differences between the predicted and actual observations.

$$\begin{aligned} \#\left( 4 \right) \end{aligned}$$

Stability metric mainly uses the mean absolute percentage error (MAPE). MAPE is a metric for quantifying the average percentage difference between a model’s predicted values and the actual observed values, serving as an indicator of the accuracy of the model’s predictions. The value of MAPE is always a percentage, making it easily interpretable across different scales. This metric ranges from 0 to positive infinity. A value approaching 0 indicates minimal prediction error and better model stability. Generally, a MAPE below 10% suggests high predictive accuracy; a MAPE between 10% and 20% indicates acceptable precision; while a MAPE above 20% signifies suboptimal performance, requiring further model optimization to improve accuracy. MAPE is calculated by finding the average of the absolute percentage differences between the predicted values and the actual observed values.

$$\begin{aligned} \#\left( 5 \right) \end{aligned}$$
