# Supplementary material for: A Deep Learning Framework for Using Search Engine Data to Predict Influenza-Like Illness and Distinguish Epidemic and Nonepidemic Seasons: Multifeature Time Series Analysis
Source: J Med Internet Res. 2025 Aug 11;27:e71786. doi: 10.2196/71786 (PMC12338964; doi:10.2196/71786)
Supplement: Multimedia Appendix 3 [file jmir-v27-e71786-s003.docx]

**Multimedia Appendix 3 Correlations between Baidu index and ILI% in all-time period at different lags**

| **Category and Terms in Chinese** | **Terms in English** | **Cross Correlation coefficient** | | |
| --- | --- | --- | --- | --- |
| **Influenza Essential Fact** |  | Lag 1 | Lag 2 | Lag3 |
| 流感传播途径 | Influenza transmission route | 0.479 | 0.506 | 0.508 |
| 流行性感冒 | Influenza | 0.640 | 0.598 | 0.528 |
| 流感 | flu | 0.388 | 0.342 | 0.261 |
| 病毒性流感 | Viral influenza | 0.344 | 0.344 | 0.328 |
| 季节性流感 | Seasonal influenza | 0.551 | 0.483 | 0.425 |
| 流感病毒 | Influenza virus | 0.561 | 0.516 | 0.437 |
| 流感抗原 | Influenza antigen | 0.294 | 0.137 | 0.056 |
| 流感并发症 | Influenza complications | 0.521 | 0.459 | 0.367 |
| 小儿感冒 | Common cold in children | 0.511 | 0.544 | 0.558 |
| 鼻炎 | Rhinitis | 0.337 | 0.365 | 0.396 |
| 气管炎 | Tracheitis | 0.128 | 0.130 | 0.186 |
| 咽炎 | Pharyngitis | 0.259 | 0.186 | 0.101 |
| 甲型流感 | Influenza A | 0.444 | 0.436 | 0.397 |
| 流感流行 | Influenza epidemic | 0.452 | 0.439 | 0.415 |
| 流感和普通感冒的区别 | Difference between influenza and common cold | 0.250 | 0.243 | 0.226 |
| 流感嗜血杆菌 | Haemophilus influenzae | 0.418 | 0.440 | 0.448 |
| 乙型流感 | Influenza B | 0.463 | 0.446 | 0.418 |
| 呼吸道传染病 | Respiratory infections | 0.457 | 0.456 | 0.397 |
| 上呼吸道感染 | Upper respiratory tract infection | 0.684 | 0.622 | 0.534 |
| **Influenza Symptom** |  | Lag 1 | Lag 2 | Lag3 |
| 发烧 | Fever | 0.398 | 0.231 | 0.104 |
| 咳嗽 | Cough | 0.438 | 0.380 | 0.254 |
| 头痛 | Headache | 0.337 | 0.380 | 0.429 |
| 咽痛 | Sore throat | 0.616 | 0.495 | 0.382 |
| 胸闷 | Chest distress | 0.251 | 0.225 | 0.157 |
| 打喷嚏 | Sneeze | 0.274 | 0.309 | 0.364 |
| 乏力 | Fatigue | 0.376 | 0.321 | 0.263 |
| 鼻塞 | Stuffy nose | 0.345 | 0.237 | 0.065 |
| 全身酸痛 | Body ache | 0.216 | 0.187 | 0.148 |
| 呕吐 | Vomiting | 0.015 | 0.030 | 0.012 |
| 呼吸困难 | Dyspnea | 0.026 | 0.020 | 0.057 |
| 嗜睡 | Drowsiness | 0.419 | 0.407 | 0.374 |
| 四肢无力 | Weakness in the limbs | 0.162 | 0.200 | 0.240 |
| 头晕 | Dizzy | 0.114 | 0.069 | 0.013 |
| 干咳 | Dry cough | 0.285 | 0.209 | 0.119 |
| 怕冷 | Fear of cold | 0.075 | 0.114 | 0.181 |
| 恶心 | Nausea | 0.367 | 0.370 | 0.372 |
| 痰多 | Phlegm | 0.086 | 0.103 | 0.137 |
| 腹泻 | Diarrhoea | 0.141 | 0.061 | 0.005 |
| 食欲减退 | Anorexia | 0.165 | 0.174 | 0.160 |
| 流感症状 | Influenza symptom | 0.476 | 0.375 | 0.305 |
| 流鼻涕 | Runny nose | 0.473 | 0.480 | 0.487 |
| **Influenza Treatment** |  | Lag 1 | Lag 2 | Lag3 |
| 感康 | Compound amantadine | 0.458 | 0.305 | 0.191 |
| 感冒清热颗粒 | Ganmaoqingrekeli | 0.458 | 0.316 | 0.189 |
| 奥司他韦颗粒 | Oseltamivir phosphate capsules | 0.482 | 0.498 | 0.469 |
| 白加黑 | White and black granules | 0.163 | 0.022 | -0.099 |
| 泰诺 | Tylenol | 0.309 | 0.156 | 0.062 |
| 流感治疗 | Influenza treatment | 0.376 | 0.335 | 0.281 |
| 连花清瘟 | Lianhuaqingwen capsule | 0.304 | 0.186 | 0.114 |
| 流感丸 | Liuganwan | 0.499 | 0.384 | 0.285 |
| 999感冒灵 | Ganmaolingkeli (999) | 0.455 | 0.356 | 0.277 |
| 康泰克 | Compound pseudoephedrine HCl sustained release capsules | 0.113 | -0.033 | -0.131 |
| 柴胡颗粒 | Chaihukeli | 0.285 | 0.194 | 0.106 |
| 板蓝根颗粒 | Banlangenkeli | 0.481 | 0.336 | 0.233 |
| 双黄连口服液 | Shuanghuanglian ral liquid | 0.160 | 0.044 | 0.027 |
| 清开灵颗粒 | Qingkailingkeli | 0.066 | -0.051 | -0.147 |
| 抗病毒口服液 | Kangbingdukoufuye | 0.503 | 0.347 | 0.235 |
| 快克 | Kuaike capsules | 0.370 | 0.220 | 0.087 |
| 抗生素 | Antibiotic | -0.266 | -0.304 | -0.330 |
| 维c银翘片 | WeiCyinqiaopian | 0.278 | 0.153 | 0.048 |
| 维生素 | Vitamin | 0.091 | 0.051 | 0.041 |
| 菊花茶 | Chrysanthemum tea | -0.472 | -0.477 | -0.470 |
| 葱白水 | Fistular onion stalk in water | -0.060 | -0.165 | -0.234 |
| 藿香正气水 | Huoxiang zhengqi oral liquid | 0.176 | 0.120 | 0.068 |
| 金银花口服液 | Honeysuckle oral liquid | 0.080 | 0.009 | -0.023 |
| 鱼腥草 | Heartleaf houttuynia herb | -0.138 | -0.151 | -0.165 |
| 退烧药 | Antipyretics | 0.524 | 0.449 | 0.393 |
| 输液 | Transfusion | 0.378 | 0.369 | 0.332 |
| 阿莫西林 | Amoxicillin | 0.679 | 0.601 | 0.494 |
| 甲流治疗 | Influenza A treatment | 0.316 | 0.320 | 0.283 |
| 甲型h1n1流感诊疗方案 | Influenza A (h1n1) diagnosis and treatment program | 0.234 | 0.241 | 0.215 |
| **Influenza Prevention** |  | Lag 1 | Lag 2 | Lag3 |
| 口罩 | Mask | 0.266 | 0.220 | 0.179 |
| 流感疫苗 | Vaccination of influenza | 0.286 | 0.274 | 0.239 |
| 流感抵抗力 | Influenza resistance | 0.389 | 0.283 | 0.160 |
| 流感疫苗副作用 | Influenza vaccine side effects | 0.068 | 0.043 | 0.006 |
| 流感预防 | Influenza prevention | 0.373 | 0.349 | 0.296 |
| 流感防控 | Influenza prevention and control | 0.520 | 0.491 | 0.462 |
| 流感隔离 | Influenza isolation | 0.395 | 0.372 | 0.346 |
| 姜糖水 | Ginger syrup | -0.011 | -0.116 | -0.220 |
| 流感疫苗接种 | Influenza vaccine vaccination | -0.424 | -0.403 | -0.395 |
| 甲型h1n1流感疫苗 | Influenza A h1n1 vaccination | 0.329 | 0.342 | 0.299 |
| 甲型h1n1流感预防 | Influenza A h1n1 prevention | 0.043 | 0.074 | 0.093 |
| 流感疫苗的最佳接种时间 | The best time to get the influenza vaccination | 0.334 | 0.341 | 0.331 |
| 流感疫苗价格 | Influenza vaccination prices | 0.371 | 0.366 | 0.332 |
| 流感疫苗接种后产生抗体 | Antibody production after influenza vaccination | 0.193 | 0.244 | 0.249 |
| 流感疫苗有必要接种 | Influenza vaccination is necessary | 0.246 | 0.234 | 0.220 |
| 流感疫苗有效期 | Influenza vaccination validity period | 0.225 | 0.279 | 0.259 |
| 三价流感疫苗 | Inactivated influenza vaccine, trivalent | 0.314 | 0.299 | 0.274 |
| 四价流感疫苗 | Inactivated influenza vaccine, quadrivalent | 0.394 | 0.400 | 0.384 |
