# Supplementary material for: A Deep Learning Framework for Using Search Engine Data to Predict Influenza-Like Illness and Distinguish Epidemic and Nonepidemic Seasons: Multifeature Time Series Analysis
Source: J Med Internet Res. 2025 Aug 11;27:e71786. doi: 10.2196/71786 (PMC12338964; doi:10.2196/71786)
Supplement: Multimedia Appendix 4 [file jmir-v27-e71786-s004.docx]

**Multimedia Appendix 4 Implementation details of the Convolutional long short-term memory network (CLSTM) framework for ILI%**

| **Framework** | **Module** | **Layer** | | **Hyperparameter** |
| --- | --- | --- | --- | --- |
| Encoder | Encoder_Backbone_Baidu search index | Transformer | | input_size=(3,193) |
|  |  |  |  | output_size=(3,64) |
|  |  |  |  | num_heads=8 |
|  |  |  |  | num_layers=1 |
|  |  |  |  | hidden_dim=128 |
|  |  |  |  | dropout=0.2 |
|  |  | CNN-LSTM | Conv1d | input_size=(3,64) |
|  |  |  |  | output_size=(3,128) |
|  |  |  |  | kernel_size=9 |
|  |  |  |  | pooling=2 |
|  |  |  | LSTM | input_size=(3,128) |
|  |  |  |  | hidden_size=(3,64,2) |
|  |  |  |  | output_size=(3,64) |
|  |  |  | LSTM | input_size=(3,64) |
|  |  |  |  | hidden_size=(3,1,2) |
|  |  |  |  | output_size=(3,1) |
|  | Encoder_Backbone_ILI% | VMD | | number of sub-signals=5 |
|  |  | CNN-LSTM | Conv1d | input_size=(3,2) |
|  |  |  |  | output_size=(3,128) |
|  |  |  |  | kernel_size=9 |
|  |  |  | LSTM | input_size=(3,128) |
|  |  |  |  | hidden_size=(3,128,2) |
|  |  |  |  | output_size=(3,128) |
|  |  |  | LSTM | input_size=(3,128) |
|  |  |  |  | hidden_size=(3,1,2) |
|  |  |  |  | output_size=(3,1) |
|  | Encoder_Fusion | GAF | | input_size=(3,2) |
|  |  |  |  | output_size=(3,1) |
| Decoder | Decoder_CNN-LSTM | CNN-LSTM | Conv1d^a^ | input_size=(3,1) |
|  |  |  |  | output_size=(3,128) |
|  |  |  |  | kernel_size=9 |
|  |  |  | Conv1d^b^ | input_size=(3,128) |
|  |  |  |  | output_size=(3,128) |
|  |  |  |  | kernel_size=3 |
|  |  |  | Conv1d^c^ | input_size=(3,128) |
|  |  |  |  | output_size=(3,128) |
|  |  |  |  | kernel_size=1 |
|  |  |  | LSTM | input_size=(3,128) |
|  |  |  |  | output_size=(3,128) |
|  | Decoder_MLP | MLP | | input_size=(3,128) |
|  |  |  |  | output_size=(3,1) |
| Optimizer | Adam | | | batch_size=64 |
|  |  |  |  | learning_rate=1e-4 |
|  |  |  |  | epochs=350 |

Note: a denotes CNN1 (first convolutional layer), b denotes CNN2 (second convolutional layer), and c denotes CNN3 (third convolutional layer). Conv1d denotes one-dimensional convolution, i.e., 1D-CNN; Adam denotes the Adaptive Moment Estimation (Adam) optimisation algorithm.
