# Supplementary figures and images for: A Deep Learning Framework for Using Search Engine Data to Predict Influenza-Like Illness and Distinguish Epidemic and Nonepidemic Seasons: Multifeature Time Series Analysis
Source: J Med Internet Res. 2025 Aug 11;27:e71786. doi: 10.2196/71786 (PMC12338964; doi:10.2196/71786)

**Multimedia Appendix 5 SHAP analysis**


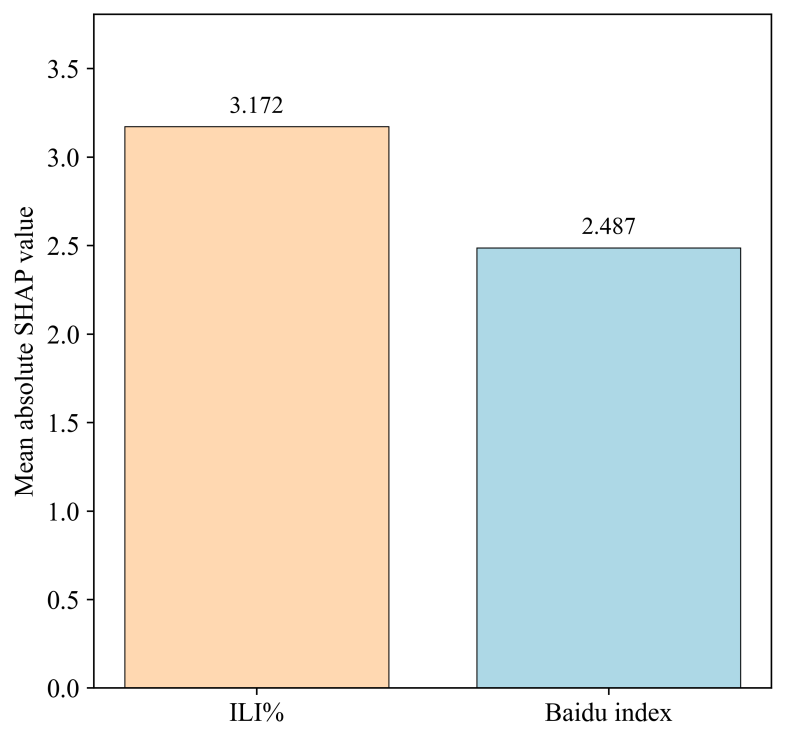


**Figure S1** SHAP analysis for CLSTM.

Supplement: Multimedia Appendix 5 [file jmir-v27-e71786-s005.docx]

**Multimedia Appendix 7. Convergence analysis**


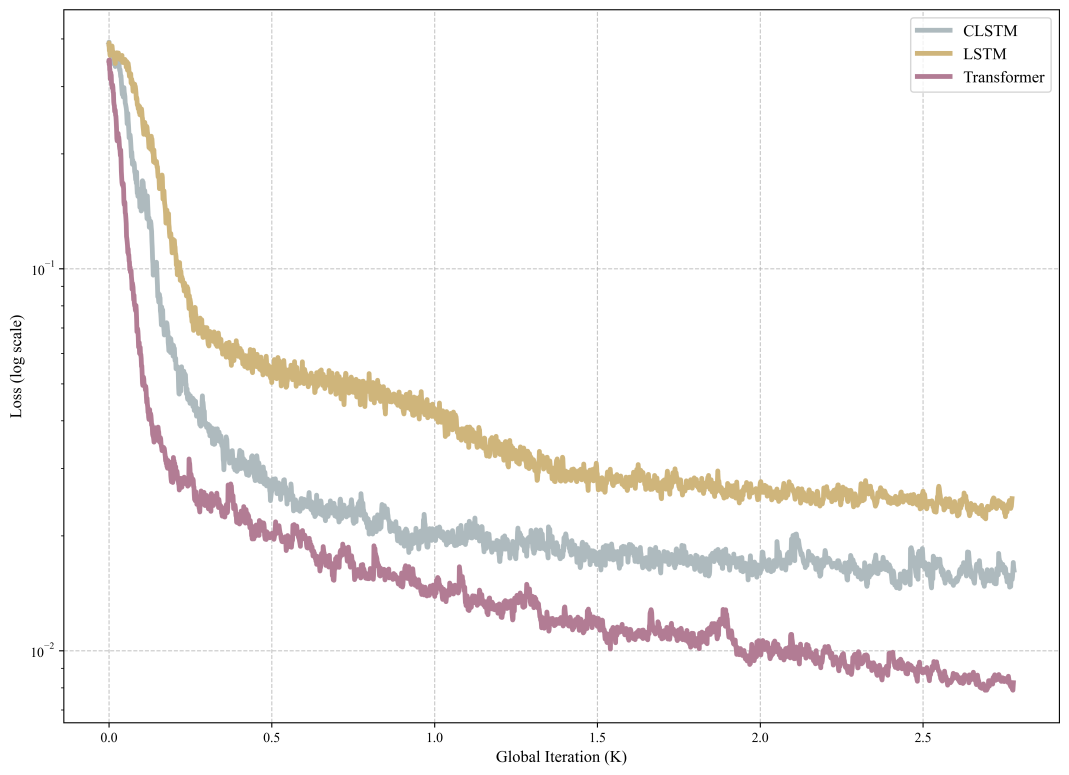


**Figure S1.** Convergence analysis for all-time period.

Supplement: Multimedia Appendix 7 [file jmir-v27-e71786-s007.docx]
