# Supplementary material for: A Deep Learning Framework for Using Search Engine Data to Predict Influenza-Like Illness and Distinguish Epidemic and Nonepidemic Seasons: Multifeature Time Series Analysis
Source: J Med Internet Res. 2025 Aug 11;27:e71786. doi: 10.2196/71786 (PMC12338964; doi:10.2196/71786)
Supplement: Multimedia Appendix 6 [file jmir-v27-e71786-s006.docx]

**Multimedia Appendix 6 Derivation process and results of Big-O complexity estimation**

**1 LSTM Big-O Complexity Estimation Derivation and Results**

The number of LSTM layers (num_layer=2) in this study is a constant. It does not affect the asymptotic complexity. Therefore, the following derivation uses a single-layer (num_layer=1) LSTM as an example.

**1.1 Symbol Definitions**

T: Input sequence length (number of timesteps); d: Input feature dimension; h: Hidden state dimension (typically set as *h=d*); W: Weight matrix; b: Bias vector.

**1.2 Single Timestep Computation Breakdown for LSTM**

Each timestep of LSTM includes the following four gating operations (using the forget gate as an example, other gates have the same structure):

(1) Forget Gate:

$$f_{t}=\sigma(W_{f}\cdot[x_{t},h_{t-1}]+b_{f})$$

Input concatenation: Current input *x_t_* (dimension *d*) and previous hidden state *h_t-1_* (dimension *h*) are concatenated into a vector [*x_t_*, *h_t-1_*] (dimension *d+h*). Matrix multiplication: Weight matrix *W_f_* has dimensions *h*×(*d+h*). The computation cost is *h*×(*d+h*). Bias addition: O(*h*) (negligible). Sigmoid activation: O(*h*) (negligible). Total computation: O*(h*×*(d+h)*).

(2) Input Gate, Candidate Memory, and Output Gate:

These have the same structure as the forget gate. Each requires O(*h*×(*d+h*)).

(3) State Update:

Element-wise multiplication and addition: O(*h*) (negligible).

**1.3 Total Complexity per Timestep**

Total computation for four gates (assuming *h=d*):

Single-step computation = 4×O(*d*×(*d*+*d*)) = O(4*d*×2*d*) = O(8*d²*) ≈ O(*d²*).

**1.4 Overall Complexity**

LSTM must process the sequence sequentially timestep by timestep:

Total complexity = T × single-step complexity = O(*T·d²*).

**2 Transformer Big-O Complexity Estimation Derivation and Results**

**2.1 Self-Attention Layer**

(1) Q/K/V Projections: Three *d*×*d* matrix multiplications. Computation cost: 3*T·d^2^* (each token’s projection complexity is *d²*, for *T* tokens).

(2) Attention Matrix:

(Q*·*K)^T^: *T*×*d* multiplied by *d*×*T* = O(*T²·d*);

Softmax + weighted sum: O(*T²*) (negligible);

Single-layer attention complexity:

$$Attn-layer=O(T^{2}\cdot d+T\cdot d^{2})$$

**2.2 Feed-Forward Layer**

Two fully connected layers: *T*×*d*×*d_ff_* + *T*×*d_ff_*×*d* ≈ O(2*T$\cdot$d$\cdot$d_ff_*). Typically *d_ff_*=4*d*, so simplified to O(*T$\cdot$d²*).

**2.3 Overall Complexity**

$$\text{Transformer=N×(Attn-layer+FFN)}=O(N\cdot T^{2}\cdot d)$$

**3 CLSTM Big-O Complexity Estimation Derivation and Results**

**3.1 Data Preprocessing**

**3.1.1 Data Input Window**

(1) Input data size: Shape (*n*, *m*), where *n* is sample count and *m* is feature count.

(2) Window size: *w*, stride length: *stride*.

(3) Number of output windows: num_samples=$\frac{n-w}{stride}+1$.

(4) Complexity analysis:

①Each window extraction operation takes O(*w$\cdot$m*).

②The time complexity of each extraction operation is O(*w**·m*)。

③Total time complexity is：

$$O\left( \frac{n-w}{stride}\cdot w\cdot m \right)\approx O\left( \frac{n\cdot w\cdot m}{stride} \right)$$

**3.1.2 Input Matrix**

(1) Input data size: Shape (*n*, *m*, *k*), where *n* is sample count, *m* is feature count and *k* is sequence length.

(2) Complexity analysis:

①The complexity of *sequence_to_square_matrix* mainly comes from the filling and reshaping operations, with an overall complexity of O(*k*).

②sequence_to_matrix is applied to each feature of each sample.

③Total time complexity is: O(*n·m·k*).

**3.1.3 Prediction Data Preprocessing**

PredData operations include data loading, sliding window generation, normalization, and VMD decomposition:

(1)VMD decomposition complexity: O(*L·K²*), where L is sequence length and *K* is mode count.

(2)Total PredData complexity:

①Data loading and window generation: O(*n·m·w* / *stride*).

②VMD decomposition: O(*L·K^2^*)

③Normalization and matrix conversion: O(*n·m·k*)

④Overall complexity is:

$$O\left( \frac{n\cdot m\cdot w}{stride}+L\cdot K^{2}+n\cdot m\cdot k \right)$$

**3.2 Model Training**

(1) Input data size: Shape (n_train_, m, k), where n_train_ is the number of training samples.

(2) Model complexity:

①Convolutional layer complexity: O(*n_train_$\cdot$m$\cdot$k$\cdot$f*), where *f* is kernel size.

②LSTM layer complexity: O(n_train_·m·k·h), where *h* is hidden units.

③Total training complexity per epoch:

$$O\left( n_{train}\cdot m\cdot k\cdot(f+h) \right)$$

(3)For E epochs:

$$O\left( E\cdot n_{train}\cdot m\cdot k\cdot(f+h) \right)$$

**3.3 Model Prediction**

(1) Input data size: Assume that the size of the test data is (n_test_, m, k), where n_test_ is the number of test samples.

(2) Prediction complexity:

$$O\left( n_{test}\cdot m\cdot k\cdot(f+h) \right)$$

**3.4 Overall Complexity**

(1) Data preprocessing:

$$O\left( \frac{n\cdot m\cdot w}{stride}+L\cdot K^{2}+n\cdot m\cdot k \right)$$

1. Model training:

$$O\left( E\cdot n_{train}\cdot m\cdot k\cdot(f+h) \right)$$

2. Model prediction:

Assuming$n_{train}\approx n_{test}\approx n,$ the overall complexity is:

$$O\left( \frac{n\cdot m\cdot w}{stride}+L\cdot K^{2}+n\cdot m\cdot k+E\cdot n\cdot m\cdot k\cdot(f+h)+n\cdot m\cdot k\cdot(f+h) \right)$$

Simplified:

$$O\left( \frac{n\cdot m\cdot w}{stride}+L\cdot K^{2}+(E+2)\cdot n\cdot m\cdot k\cdot(f+h) \right)$$

1. Overall Complexity

The overall complexity of CLSTM is polynomial-level, mainly consisting of the complexity of data preprocessing, VMD decomposition, model training, and prediction. Among these, the complexity of model training, O(*E$\cdot$n$\cdot$m$\cdot$k*$\cdot$(*f* + *h*)), is the dominant part, especially in the case of large-scale data and multiple rounds of training.
